# Absence of amyloid β oligomers at the postsynapse and regulated synaptic Zn^2+^ in cognitively intact aged individuals with Alzheimer’s disease neuropathology

**DOI:** 10.1186/1750-1326-7-23

**Published:** 2012-05-28

**Authors:** Nicole L Bjorklund, Lindsay C Reese, V-M Sadagoparamanujam, Valeria Ghirardi, Randall L Woltjer, Giulio Taglialatela

**Affiliations:** 1 Department of Neuroscience and Cell Biology, Galveston, TX, 77555, USA; 2 Department of Preventive Medicine and Community Health, University of Texas Medical Branch, Galveston, TX, 77555, USA; 3 Department of Pathology, Oregon Health & Science University, Portland, OR, 97201, USA

**Keywords:** Aβ oligomers, Alzheimer’s disease, hippocampus, asymptomatic, zinc, synaptic vesicle

## Abstract

**Background:**

Early cognitive impairment in Alzheimer Disease (AD) is thought to result from the dysfunctional effect of amyloid beta (Aβ) oligomers targeting the synapses. Some individuals, however, escape cognitive decline despite the presence of the neuropathologic features of AD (Aβ plaques and neurofibrillary tangles). We term this group Non-Demented with AD Neuropathology or NDAN. The present study illustrates one putative resistance mechanism involved in NDAN cases which may suggest targets for the effective treatment of AD.

**Results:**

Here we describe the localization of Aβ oligomers at the postsynapse in hippocampi from AD cases. Notably, however, we also found that while present in soluble fractions, Aβ oligomers are absent from hippocampal postsynapses in NDAN cases. In addition, levels of phosphorylated (active) CREB, a transcription factor important for synaptic plasticity, are normal in NDAN individuals, suggesting that their synapses are functionally intact. Analysis of Zn^2+^ showed that levels were increased in both soluble fractions and synaptic vesicles in AD hippocampi, paralleled by a decrease of expression of the synaptic vesicle Zn^2+^ transporter, ZnT3. Conversely, in NDAN individuals, levels of Zn^2+^ in soluble fractions were significantly lower than in AD, whereas in synaptic vesicles the levels of Zn^2+^ were similar to AD, but accompanied by preserved expression of the ZnT3.

**Conclusions:**

Taken together, these data illustrate that despite substantial AD neuropathology, Aβ oligomers, and increased synaptic vesicle Zn^2+^, susceptible brain tissue in these aged NDAN individuals features, as compared to symptomatic AD subjects, significantly lower total Zn^2+^ levels and no association of Aβ oligomers with the postsynapse, which collectively may promote the maintenance of intact cognitive function.

## Background

There is no effective treatment currently available for Alzheimer’s disease (AD), the most common and severe age-related dementia, and the number of cases diagnosed each year is rising [1]. New ideas and effective therapeutic targets are therefore urgently needed. Neuropathologically, AD is characterized by the presence in the central nervous system of extracellular senile plaques primarily formed by deposits of large fibrillar aggregates of the amyloid beta (Aβ) protein and by intracellular neurofibrillary tangles (NFT) formed by hyper-phosphorylated tau protein [2,3].

However, aged individuals with abundant Aβ plaques and NFT who are nonetheless cognitively intact have also been described [4-8]. Notably, the National Institute of Health/National Institute on Aging and the Alzheimer’s Association have recently included such individuals in their joint official guidelines for neuropathologic assessment of AD and classified them as individuals who have AD neuropathologic changes in the absence of cognitive impairment [9]. It is currently unclear why these individuals, who we term Non-Demented with Alzheimer’s Neuropathology (NDAN), are resistant to the clinical manifestations of AD despite a significant burden of pathological lesions equivalent to what is normally found in comparably aged subjects with established AD. In one study, NDAN individuals have been found to have larger hippocampal and total brain volume [6], suggesting cognitive reserve may play a role, but evidence remains incomplete. Nonetheless, the now recognized existence of NDAN individuals suggests that there are mechanisms by which the aging human brain may cope with cognitive dysfunction brought about by Aβ and NFT’s; and determining the protective molecular mechanisms involved in these resistant individuals could lead to the identification of novel targets for the development of effective therapeutic approaches [9].

In recent years, the focus of research on the toxic role of Aβ has shifted from the Aβ fibrils that comprise the insoluble plaques, to the smaller, soluble oligomeric Aβ aggregates that precede plaque formation. These oligomers are considered to be the most toxic Aβ species [10,11]. In experiments using cultured cells, Aβ oligomers prepared from synthetic peptides are cytotoxic whereas Aβ monomers or fibrils are relatively innocuous [12,13]. Furthermore, Aβ oligomers of human and murine origin have been shown to induce memory deficits when injected intracerebroventricularly in rodents [14-16] and to localize to synapses [17,18], where they affect the protein composition of the postsynaptic density (PSD) [17,19] and impair synaptic plasticity [16,20]. The downstream effects of Aβ oligomer synaptotoxicity include disruption of intracellular Ca^2+^ homeostasis [13] and subsequent calcineurin-dependent dephosphorylation of NFAT [21] and key functional synaptic proteins such as the cAMP response element binding (CREB) protein [12], a transcription factor regulating the transcription of genes involved in synapse maintenance and formation [22].

Zn^2+^, a transition metal dysregulated in AD [23,24], has been shown to stabilize Aβ oligomers [25] and target them to the postsynapse [26]. Zn^2+^ is an important neuromodulator involved in many processes in the brain. Many studies have found alterations in Zn^2+^ levels in the AD brain, with most reporting an increase in its concentration [27-29]. In addition, the mRNA and protein expression of the Zn^2+^ transporter 3 (ZnT3) and the Zn^2+^ binding protein, metallothionein 3, are decreased in AD [30-33]. This collective evidence indicates that the regulation of Zn^2+^ is unbalanced in the AD brain and suggests a role of Zn^2+^ in AD pathogenesis.

Zn^2+^ can be taken up into presynaptic terminals and then packaged into synaptic vesicles along with glutamate by the vesicle-specific ZnT3 [34]. During synaptic transmission, Zn^2+^ and glutamate are co-released into the synaptic cleft and interact with postsynaptic proteins [35,36]. Zn^2+^ is essential for the modulation of calcium channels, AMPA receptors, and NMDA receptors [37-39]. Therefore, changes in levels of synaptic Zn^2+^ have the potential to alter synaptic transmission. Furthermore, Zn^2+^ can coordinate with the N-terminal region of Aβ most likely by binding with three histidines and an oxygen side chain [40-42]. This induces conformational changes and facilitates intermolecular interactions conducive to oligomer formation [43-45]. Indeed, the coordination of Zn^2+^ interferes with Aβ aggregation process, preventing fibril assembly and favoring the formation of stable oligomers [25]. These molecular events are induced with transient Zn^2+^ pulses, similar to those that occur during synaptic release, which has the potential of stabilizing ambient, inter-synaptic oligomeric Aβ [25]. In addition to stabilizing oligomers, Zn^2+^ enhances the synaptic association of Aβ as synaptic targeting of Aβ is decreased by Zn^2+^ chelation or in primary neuronal cell cultures from ZnT3 knockout mice that have virtually no synaptic Zn^2+^[26]. This collective evidence highlights the role of releasable Zn^2+^in both the toxicity and targeting of Aβ oligomers within the synapse.

On these bases, the cognitive integrity of NDAN individuals suggests a lack of synaptic dysfunction that may reflect resistance to the synaptotoxic effects of Aβ oligomers and dysregulated Zn^2+^. The focus of the present study was to investigate these features in the hippocampus from cognitively-intact NDAN subjects as compared to AD individuals who succumb to dementia. We performed subcellular fractionations coupled with immunoblotting and graphite furnace-atomic absorption spectrophotometry (GF-AAS) to measure Zn^2+^ along with immunohistochemical analyses of human hippocampi to compare age-matched control and AD to NDAN cases. We found substantial differences between the AD and NDAN samples in terms of Aβ oligomer presence at the PSD, integrity of synaptic proteins, and Zn^2+^ regulation. These data suggest details about toxic mechanism(s) in AD that initiate cognitive decline and suggest therapies that target Zn^2+^ homeostasis in the brain may have efficacy in the prevention of cognitive decline in AD.

## Results

### Case descriptions

The neuropathological and clinical characteristics of the cases used in this study are summarized in Table 1. The details of the cognitive examination of the study subjects have been reported previously [4,6,46] and neuropathological methods are detailed in the Methods section. Groups were designated based on Braak and plaque stages according to CERAD specifications and the Mini Mental State Exam (MMSE) test scores as described in the Methods section. The control group had Braak stage 0–2 and no more than sparse neuritic plaques and were cognitively intact on annual neuropsychiatric testing, as summarized by terminal MMSE scores >25. The AD group had Braak scores of 6 and moderate to frequent neuritic plaques with below normal MMSE scores (average of 9). The NDAN cases had Braak scores that ranged from 4 to 6 and a range of neuritic plaque densities, but typically with moderate densities of neuritic plaques. The MMSE scores in this group were comparable to those in age-matched normal individuals (>25) with an average of 28. The postmortem interval (PMI) was <24 h, except for two cases. Tissue morphology was well preserved in all samples and there was no sign of protein degradation in Western blots performed on protein extracts from samples used in this study.

**Table 1:** Characteristics of cases defined by neuropathology and dementia status.

**Diagnosis**	**n**	**Age (yrs) (mean)**	**Sex**	**PMI (hr)**	**Braak (median)**	**Plaque (median)**	**MMSE (mean)**
**Control**	13	84.8	8 F, 5 M	14.2 ± 3	1	1	29
**AD**	21	81	12 F, 8 M, 1NA	12.2 ± 2	6	3	9
**NDAN**	10	89.5	7 F, 3 M	10.7 ± 4	5	3	28

PMI: Postmortem interval; NA: data not available.

### Immunohistochemical, neuropathological, and Aβ assessments demonstrate the similarities between AD and NDAN

**Figure 1:**
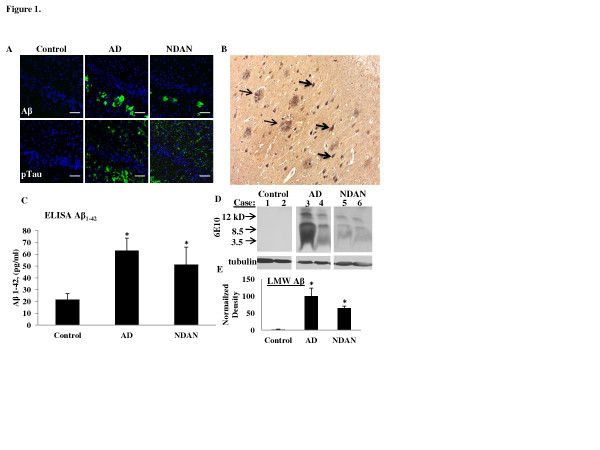
**Pathological signatures of AD occur in cognitively intact individuals (NDAN).** (**A**) Immunohistochemical detection shows reactivity for Aβ plaques (top row) and phosphorylated tau (bottom row) in the dentate gyrus of individuals classifed as control (Braak 1, Plaque 3, MMSE 30), AD (Braak 6, Plaque 1, MMSE 2), or NDAN (Braak 6, Plaque 1, MMSE 27). DAPI-containing mounting medium was used to visualize nuclei (blue). 20x magnification, scale bar 100 μm. (**B**) Bielschowsky staining in the hippocampus of an NDAN individual shows plaques (thin arrows) and neurofibrillary tangles (thick arrows). (**C**) An ELISA shows increased Aβ_1-42_ in AD and NDAN hippocampi (n = 3 per group and asterisks (*) denote values significantly higher than control with *p* < 0.05). (**D**) Representative Western blot of soluble hippocampal fractions (300 μg) from individual cases probed with 6e10 shows that LMW Aβ species are present in both AD and NDAN. Densitometric analysis (**F**) of the major bands (monomer at 4kD and dimer at 8.5kD) together shows that AD and NDAN cases are significantly different than control where the average optical density is set at 100; the asterisks (*) denote statistical significance compared to the control value (*p* = 0.022, ANOVA); The Western blot shown is representative of 9–10 individual cases assayed in each group and experiments were repeated 3 – 4 times

The pathological analysis described above for AD and NDAN cases confirmed that these two groups have a comparable presence of Aβ plaques and NFTs, further corroborating the notion that cognitive diversity between AD and NDAN individuals could not be ascribed to different extents of these two AD-related neuropathological features [5]. Figure 1 shows representative panels of the immunohistochemical detection of Aβ and phosphorylated tau (Figure 1A) and Bielschowsky staining of amyloid plaques and NFTs (Figure 1B) in the hippocampus from representative cases used in the present study. Furthermore, the levels of Aβ_1-42_ were equally increased in NDAN and AD as determined by a solid phase sandwich enzyme-linked immunosorbent assay (ELISA) (Figure 1C).

In addition to soluble monomers, Aβ_1-42_ is normally present in the affected human brain in structurally distinct aggregated forms, including large fibrils, which are the main component of senile plaques and small soluble oligomers, which are believed to be the most neurotoxic Aβ species [47-49], We therefore first determined which of these low molecular weight (LMW) Aβ oligomeric species were present in the hippocampus of NDAN cases as compared to AD cases. Western blot analysis revealed that LMW Aβ species of ~4, 8, and 12 kDa were detectable in soluble fractions from hippocampi of both AD and NDAN cases (Figure 1D). Statistical analysis applied to densitometry values obtained following this approach showed that the levels of LMW Aβ species were significantly increased in both groups compared to control (Figure 1E). Thus, the presence of LMW Aβ species in the NDAN samples eliminates the possibility that these individuals remain cognitively intact due to lack of highly neurotoxic Aβ oligomers.

These data highlight the presence of comparable levels of plaques, tangles, Aβ _1–42_ levels, and LMW Aβ oligomers between AD and NDAN.

### Low molecular weight Aβ oligomers are highly associated with the postsynaptic density in AD, but not NDAN cases

**Figure 2:**
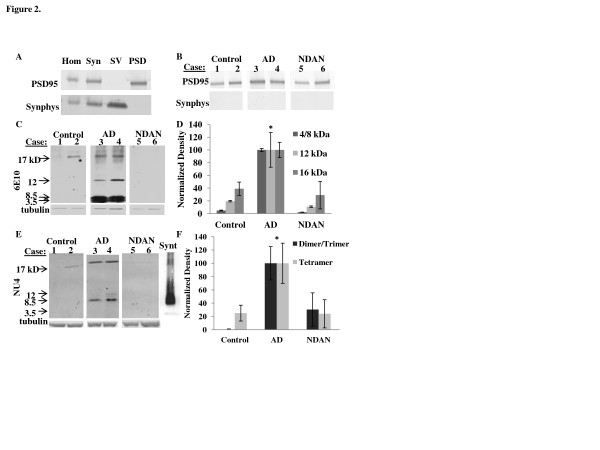
**Synaptic fractionation demonstrates that low molecular weight Aβ oligomers associate with the PSD in AD but not NDAN hippocampal specimens.** (**A**) PSD95, a postsynaptic marker, and synaptophysin (Synphys), a presynaptic marker, were probed in the total homogenate (Hom), synaptosomal (Syn), synaptic vesicles (SV), and postsynaptic density (PSD) fractions isolated from a frozen human hippocampal specimen from a control, non-demented aged individual. Enrichment of the different fractions is shown by the presence/absence of synaptic markers. The fractionation was successful for all case types (control, AD and NDAN) as shown by the purity of PSD fractions in (**B**). The isolated PSD fraction from hippocampal samples from different cases from each group (aged matched controls, AD, and NDAN) were immunoblotted together (representative blot shown; 40 μg protein each lane of a 10-20% tris-glycine gradient gel) and probed using 6e10 (**C**) showing LMW Aβ only associates with AD samples. (**D**) Densitometric analyses of each of the Aβ species (monomer/dimer = 4–8.5 kD band, trimer = 12 kD, and tetramer = 16 kD) demonstrates that all Aβ species are increased in AD. The results are expressed as the mean ± SEM using propagation of error and normalized with AD = 100%. The asterisk denotes the values significantly higher than control (monomer/dimer, *p* = 0.012, trimer, *p* = < 0.001, and tetramer, *p* = 0.014; ANOVA; a Bonferroni correction was required for the monomer/dimer and trimer density values). Hippocampus PSD fractions (80 μg) from age-matched controls, AD, and NDAN samples were immunoblotted and probed using NU4 (**E**). A lane is included at the end showing synthetic (Synt) Aβ oligomers probed by NU4 for comparison. The densitometric analysis (**F**) of the dimer/trimer band shows that AD cases are significantly higher than control and NDAN (both groups are essentially the same as background), while the tetrameric species was not found to be significantly different across the groups (*p* = 0.67). The asterisk denotes significance compared to control, *p* < 0.05, ANOVA. All Western blots shown are representative of 9–10 individual cases assayed in each group and experiments were repeated 3 – 4 times

Aβ oligomers have been shown to accumulate at the synapses where they induce dysfunctional changes that impair synaptic integrity [18,20,49]. Therefore, to further assess the localization of the LMW Aβ species detected in the AD and NDAN brains, we performed a synaptic fractionation that separates presynaptic and postsynaptic fractions [50] and probed the postsynaptic fractions for the presence of Aβ by Western blotting. Successful synaptic fractionation of the brain tissues was confirmed by probing the Western blots of the different fractions with antibodies directed against pre- and postsynaptic markers as illustrated in Figure 2A. The fractionation was successful for each group (age-matched control, AD, and NDAN) as shown by the enrichment of PSD95 in the PSD fraction (Figure 2B).

When blots were probed for Aβ, we found that LMW Aβ species were highly concentrated in the postsynaptic fractions from AD hippocampi, but completely absent from aged-matched controls and NDAN cases (Figure 2C). Densitometric analysis (Figure 2D) confirmed that LMW Aβ species were abundant at the PSD isolated from AD hippocampi but absent (undetectable above background) in NDAN cases. This corroborates previous reports that Aβ selectively targets the PSD in AD [17,51].

To confirm that the LMW Aβ species found at the PSD from AD hippocampi were oligomers, an Aβ oligomer-specific antibody, NU4, was utilized [52]. The representative Western blot in Figure 2E demonstrates that the low molecular Aβ species detected in AD samples are indeed oligomers (particularly dimers and trimers, as assessed by comparison to synthetic (Synt) Aβ oligomers). On the other hand, neither dimeric nor trimeric Aβ species were detectable in the NDAN samples, and this was statistically confirmed by densitometric analysis of the blots (Figure 2F). Interestingly, an Aβ tetrameric species was present in the sample from all groups, including the control samples, which suggests that this species is not uniquely associated with clinically manifested AD. These data demonstrate that while present in the soluble fractions from both AD and NDAN cases, Aβ oligomers are highly associated with the PSD only in AD hippocampi, but are absent in the PSD prepared from NDAN cases.

### Expression of neuronal nuclear phosphorylated CREB is preserved in NDAN hippocampi

**Figure 3:**
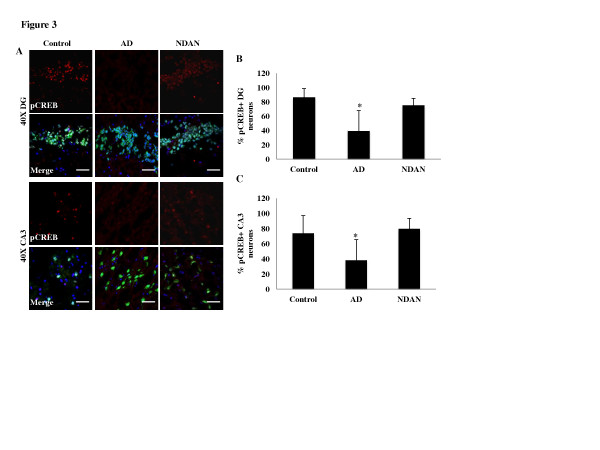
**Synaptic integrity is maintained in the NDAN hippocampus as suggested by preserved levels of neuronal nuclear pCREB.** (**A**) pCREB (red) and NeuN (green) expression was assessed in 10 μm sections of hippocampus. DAPI-containing mounting medium was used to visualize nuclei (blue). pCREB immunoreactivity was decreased in the DG and CA3 neurons of AD hippocampi but not in NDAN cases (scale bar 50 μm). The number of neurons positive for nuclear pCREB was counted in the (**B**) DG and (**C**) CA3. Two images per region (DG and CA3) were analyzed for each clinical case. The raw images were thresholded, and a blind counter quantified the number of neurons in the field of view, and how many of these exhibited nuclear pCREB immunoreactivity. This value is expressed as the percentage of neurons that are pCREB positive ± SEM (the asterisks denote statistical significance compared to controls at *p* ≤ 0.05; ANOVA)

Our previous studies have shown that one downstream effect of Aβ oligomers dysfunctionally impacting synapses is the decrease of active, phosphorylated CREB (pCREB), a transcription factor essential for synaptic plasticity and memory function [12,16,53]. The cognitive integrity of NDAN individuals suggests that synaptic function is preserved in these individuals, which would thus be consistent with the observed absence of Aβ at the postsynapse. We therefore determined the expression of neuronal pCREB in each of the samples using immunohistochemistry coupled to confocal microscopy (Figure 3A) as an index of functional synaptic integrity. When pCREB levels were quantified in granular neurons of the dentate gyrus (DG) and in pyramidal neurons of the cornu ammonis region 3 (CA3) in postmortem human hippocampus (Figure 3B) we found significantly fewer pCREB positive neurons in both cell populations in subjects with AD. Conversely, despite the presence of abundant Aβ and NFTs in the NDAN hippocampi, levels of pCREB were similar to those of control cases. Levels of pCREB were also measured by Western blot in total homogenate fractions from each group (Additional File 1). Consistent with the immunohistochemistry results, we found that there was a decrease in the AD samples, but not NDAN, which however did not reach statistical significance. This result is not completely unexpected if one considers that total homogenate does contain proteins contributed from both neurons and glial cells, both of which express CREB, thus reducing the sensitivity of Western blotting to detect changes in pCREB levels occurring solely in neurons (as our immunohistochemistry results seem to indicate). Notwithstanding this technical limitation, direct observation by immunohistochemistry and Western blot detection collectively indicate that pCREB levels in hippocampal neurons are not reduced in NDAN as compared to AD cases, thus suggesting that synaptic integrity in NDAN neurons is indeed preserved.

### Total Zn^2+^ levels, releasable Zn^2+^, and ZnT3 expression is altered in AD and NDAN

**Figure 4:**
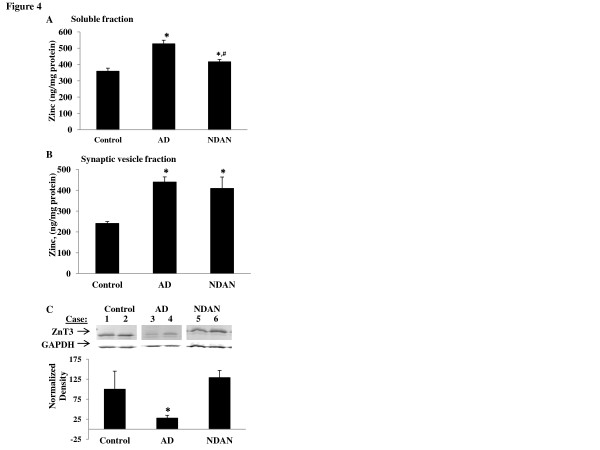
**Differentially altered levels of total Zn**^**2+**^**, releasable Zn**^**2+**^**, and ZnT3 expression in AD and NDAN hippocampus.** (**A**) Soluble hippocampal fractions were normalized for protein (500 μg), digested with hydrogen peroxide and nitric acid, and analyzed using a GF-AAS. Zn^2+^ levels in both AD and NDAN samples were increased as compared to control, but NDAN Zn^2+^ levels were significantly lower than AD levels. After a Bonferroni correction, ANOVA results show *p* = < 0.001, n = 9–10 samples per group. (**B**) Zn^2+^ analysis in synaptic vesicles shows that the Zn^2+^ concentration in AD and NDAN is significantly higher than control (*p* = < 0.001 (after Bonferroni correction), n = 6–9 per group). (**C**) Representative Western blot and densitometric analysis (**D**) of ZnT3 expression in the hippocampus of individual cases shows a significant decrease in AD, but not in NDAN samples, which is denoted by an asterisk (*t*-test, *p* < 0.001 n = 6–9 per group, repeated 4 times)

The exclusion of Aβ at the postsynapses in NDAN individuals suggests that mechanisms involved in the pathological targeting of Aβ to synapses may be altered or absent in these individuals. Among these, synaptic Zn^2+^ can coordinate with Aβ oligomers and target them to the synapse where the oligomers can induce toxic effects [26]. We therefore measured Zn^2+^ levels to determine if alterations in Zn^2+^ concentration are associated with the absence of Aβ oligomers at the postsynapse in NDAN individuals. Soluble fractions from hippocampi were processed as described in the methods and Zn^2+^ levels were analyzed using GF-AAS. Our results show that the AD specimens had significantly higher Zn^2+^ concentrations than control (Figure 4A). Zn^2+^ levels in the NDAN tissue were also higher than control, but significantly lower than those observed in AD samples.

Zn^2+^ in the soluble fractions would include both protein-bound and releasable free Zn^2+^. The releasable Zn^2+^ stored into presynaptic vesicles and released during synaptic activity becomes available to coordinate with proteins present at the synapse [54,55], including Aβ oligomers. Therefore, to achieve a better assessment of the levels of the releasable Zn^2+^ pool, synaptic vesicles were isolated for analysis of Zn^2+^ content (Figure 4B). The levels of Zn^2+^ in the synaptic vesicles were increased in both AD and NDAN samples as compared to brain samples from age-matched control individuals.

The ZnT3 transports Zn^2+^ into synaptic vesicles and is transcriptionally regulated by Zn^2+^[54]. Previously, mRNA and protein expression of this transporter was found to be decreased in AD brain [30,32]. Immunoblots probing for ZnT3 confirmed this finding and also showed that ZnT3 expression in NDAN was maintained at control levels (Figure 4C). Taken together these data suggest that despite comparable levels of vesicular Zn^2+^, but higher expression of the ZnT3 and lower Zn^2+^ in the soluble fraction, the regulation of Zn^2+^ homeostasis in NDAN individuals may be better preserved in comparison to AD cases, thus possibly reducing the Zn^2+^: Aβ interaction and subsequent Aβ targeting to the PSD.

## Discussion

**Figure 5:**
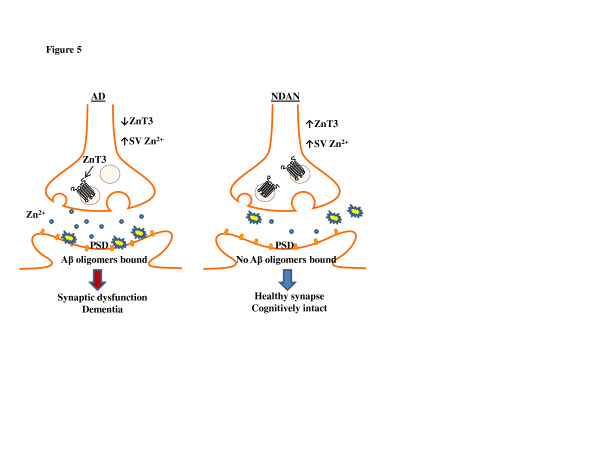
**Aβ and Zn**^**2+**^**differences in AD and NDAN hippocampal synapses**. A schematic summarizing the findings presented in this report. In AD there is increased Zn^2+^ in the soluble fraction compared to control and NDAN. However, both AD and NDAN have increased levels of Zn^2+^ in the synaptic vesicles (SV) which may actually reflect a storage compensatory mechanism to high Zn^2+^ levels. The reduction of ZnT3 in AD may reflect a dysfunction of this compensatory mechanism, allowing for total Zn^2+^ levels to increase. Zn^2+^ is necessary for stabilization and targeting of Aβ oligomers to the PSD. Aβ oligomers are highly associated with the PSD in AD hippocampal synapses, but are absent at NDAN synapses. This key difference could likely be due to the levels and regulation of synaptic Zn^2+^, resulting in synaptic dysfunction and cognitive decline in AD and preserved synapses and cognition in NDAN cases

The main goal of the present study was to investigate what molecular events are associated with the ability of some aged individuals to remain cognitively intact despite the presence of increased Aβ_1-42_, Aβ plaques and NFTs (Figrue 1). Our results show that NDAN individuals do have toxic Aβ oligomers, but that, at variance with AD subjects, these oligomers do not associate with the PSD. Along with the absence of oligomers at the PSD, pCREB levels were sustained in NDAN, which suggests that synaptic function is maintained. The total Zn^2+^ levels in NDAN were lower than those in AD, albeit still higher than control. On the other hand, synaptic vesicle Zn^2+^ was increased in both AD and NDAN, whereas ZnT3 expression was decreased in AD but preserved in NDAN samples. Taken together, and in light of the current literature evidence as detailed below, these novel results suggest that Zn^2+^ regulation may be part of a mechanism that prevents binding of Aβ oligomers to the postsynapse in NDAN individuals, thus likely contributing to preservation of synaptic and cognitive integrity (Figure 5).

The neuropathological and immunohistochemical analysis found that the extent and distribution of Aβ deposits and NFTs/hyper-phosphorylated tau in the hippocampus is comparable between AD and NDAN cases (Table 1 and Figure 1). Additionally, Aβ_1-42_ levels are increased in NDAN similar to levels in AD and Aβ species can be detected by Western blot analysis in total protein extracts from both AD and NDAN hippocampi (Figure 1D & E). The presence of LMW Aβ species demonstrates that intact cognition could not be simply ascribed to efficient, or off-pathway aggregation of Aβ resulting in low levels or absence of Aβ oligomers.

When Aβ was assayed by Western blot in purified PSD fractions the results showed a robust presence of Aβ species in the AD cases, consistent with previous studies [26,51]. However, there was a complete absence of LMW Aβ species at the PSD isolated from NDAN hippocampi (Figure 2C). These species were confirmed to be Aβ oligomers using a LMW Aβ oligomer specific antibody, NU4 (Figure 2E) [52]. Densitometric analysis confirmed that at the PSD, the level of LMW Aβ of NDAN was the same as control and indistinguishable from background. However, the presence and level of an apparent Aβ tetramer was surprisingly equal across the groups. The postsynaptic presence of this Aβ tetrameric species in the control samples suggests that it may not be relevant to processes of cognitive loss in AD. That LMW Aβ oligomers are present in the soluble fraction, but not postsynaptic fractions of NDAN cases suggests that the postsynaptic localization, rather than the mere presence of the oligomers is key in initiating a cascade of neurodysfunctional events eventually leading to cognitive decline. Indeed, naturally occurring human LMW Aβ oligomers, specifically dimers and trimers, have been shown to be synaptotoxic [20,56] and the absence of these oligomeric species at the postsynapse may negate their toxic effect, allowing synapses to remain functional.

The absence of oligomers at the PSD in the NDAN samples may thus indicate that synaptic integrity is preserved in these individuals. Indeed, in this study we found that levels of neuronal pCREB, a transcription factor required for proper synaptic plasticity and memory function [57], were unchanged in NDAN as compared to age-matched controls. Conversely, neuronal pCREB levels were significantly decreased in both the CA3 and DG in the AD hippocampus as compared to age-matched normal individuals or NDAN (Figure 3). As pCREB is essential for synaptic function, these results further the notion that synaptic functional integrity is preserved in NDAN cases, perhaps consequent to the lack of synaptic Aβ oligomers.

The absence of Aβ oligomers at the PSD in NDAN hippocampi indicates the existence of mechanisms that actively target Aβ oligomers to the synaptic compartment and suggests that such mechanisms are impaired or absent in these cognitively-intact individuals. Among possible others, the regulation of synaptic Zn^2+^ could be a likely candidate mechanism. Indeed, the most significant findings suggesting that Zn^2+^ regulation may be such a factor in AD, is the reported coordination, stabilization, and synaptic targeting of Aβ oligomers by Zn^2+^[25,26,43] along with increased Zn^2+^ levels and decreased ZnT3 mRNA and protein expression in the AD brain [27-30,32]. In addition, the coordination of Zn^2+^ with Aβ (which potentially reduces Zn^2+^ availability to target receptors and channels) produces a Zn^2+^ deficient condition, which also causes synaptic dysfunction [19]. Therefore, a double insult is produced; increased synaptic targeting along with decreased Zn^2+^ signaling.

Consistent with previous reports [27-29], we found that the total Zn^2+^ levels were increased in the AD samples (Figure 4A). We further found that the Zn^2+^ levels in the soluble fraction from NDAN samples were significantly lower than in AD samples, albeit higher than control. This latter result is not unexpected if one considers that amyloid plaques are enriched in Zn^2+^ and other metals [58,59]. The neuropathological analysis of NDAN brains showed that the plaque load was comparable to the AD samples and therefore the decreased Zn^2+^ levels in NDAN suggest that there is a mechanism capable of lowering levels of free Zn^2+^ and perhaps limiting the Zn^2+^ available to coordinate with Aβ and target it to the PSD.

The main storage area in the brain for Zn^2+^ that is not bound to proteins is synaptic vesicles [54]. The Zn^2+^ in these vesicles may be released along with glutamate following presynaptic depolarization and then interact with proteins both in the synaptic cleft and the postsynaptic membrane. Synaptically released Zn^2+^ affects the molecular behavior of ambient Aβ oligomers [25]. Therefore, the measurement of the vesicular Zn^2+^ pool provides a better understanding of Zn^2+^ regulation and homeostasis in AD. We found that synaptic vesicle Zn^2+^ concentrations were increased in AD and NDAN compared to control (Figure 4B). Increased vesicular Zn^2+^ in both AD and NDAN, along with reduced Zn^2+^ in the soluble fractions from NDAN as compared to AD, suggests the existence of a compensatory mechanism in NDAN to remove excess Zn^2+^.

Indeed, we found that the expression of the synaptic vesicle-specific Zn^2+^ transporter ZnT3 was preserved in NDAN samples at levels comparable to those observed in age-matched control cases. While our results confirm lower ZnT3 levels in AD (Figure 4C), preserved levels of ZnT3 and elevated vesicle Zn^2+^ in NDAN may represent a mechanism that contain the extent of dysregulation of Zn^2+^ homeostasis in the brain. Such regulation of Zn^2+^ may limit the targeting of Aβ oligomers to the postsynapse in NDAN, allowing for proper synaptic function and resilience to cognitive decline.

A wealth of experimental evidence strongly suggests that keeping the Aβ oligomers from associating with the synapses would reduce synaptic dysfunction and thus represent a valid approach to preserve cognitive integrity despite the presence of Aβ in the CNS of diseased individuals. Here we have shown for the first time that individuals who have significant neuropathologic features of AD, but who are cognitive intact, do not have LMW Aβ oligomers associated with the postsynapse along with evidence of a better preserved synaptic Zn^2+^ homeostasis as compared to demented AD subjects. Taken together, our results thus suggest that efficient synaptic Zn^2+^ regulation and lack of Aβ oligomers at the PSD may contribute to preserving cognitive integrity despite the presence of AD neuropathology.

However, while indicating a central role for synaptic Zn^2+^ regulation, our present results do not exclude that other mechanisms may contribute to the ability of the PSD to resist Aβ oligomer binding in the NDAN brain. To that end, it is worth noting recent evidence suggesting that sustaining insulin signaling may prevent Aβ oligomer binding to synaptic spines in cultured neurons [56], and ongoing studies in our laboratory are investigating whether such mechanisms may be also at play in the NDAN brain. Further characterization of the NDAN population may thus reveal additional details about the collective mechanism(s) by which the human brain can effectively resist the synaptic targeting of Aβ oligomers; it is prudent to argue that such mechanism(s) will represent a valid therapeutic target for cognitive preservation in the context of the pathologic processes of AD.

## Methods

### Case subjects

Frozen mid-hippocampus tissue was obtained from the Oregon Brain Bank at Oregon Health and Science University (OHSU) in Portland, OR (See Table 1). Donor subjects were enrolled and clinically evaluated in studies at the NIH-sponsored Layton Aging and AD Center (ADC) at OHSU. Subjects were participants in brain aging studies at the ADC and received annual neurological and neuropsychological evaluations, with a clinical dementia rating (CDR) assigned by an experienced clinician. Controls had normal cognitive and functional examinations. The AD subjects were diagnosed by a clinical team consensus conference, met the National Institute for Neurological and Communicative Disorders and Stroke-Alzheimer’s Disease and Related Disorder Association diagnostic criteria for clinical AD, had a CDR of greater than 1.0, and neuropathologic confirmation at autopsy (after informed consent). Tissue use conformed to institutional review board-approved protocols. Neuropathologic assessment conformed to National Institute on Aging-Reagan consensus criteria. All brain tissue was examined by a neuropathologist for neurodegenerative pathology including neurofibrillary tangles and neuritic plaques. Using standardized CERAD criteria [60], cases were assigned an amyloid score based on the deposition of amyloid plaques in the brain (0 = no plaques, 1 = sparse plaques, 2 = moderate plaques, and 3 = dense plaques), and a Braak stage (0–6; with 6 being the most severe) indicative of the level and location of hyper-phosphorylated tau tangles [3]. In addition to the pathological information detailed above, demographical data were received along with the frozen tissue. These included age, sex, and MMSE score [61] for each case.

To ensure that the variations in postmortem interval (PMI) did not affect the measurements, a correlation analysis between PMI values and results obtained in the various assays presented here was performed using a Pearson’s correlation test. No correlation was found for any of the experiments presented (Additional File 2), and therefore observed differences could not be attributed to differences in non-specific postmortem tissue degradation among the various groups.

### Immunohistochemistry

Five-mm sections of the mid hippocampus were brought out of storage at −80°C and equilibrated to −20°C before embedding in Tissue-Tek O.C.T. compound (Sakura Finetek, Torrence, CA, USA). Ten μm sections were cut and affixed to Superfrost Plus slides (Thermo Fisher Scientific, Waltham, MA, USA), for further storage as needed at −80°C. After equilibrating to room temperature, sections were rinsed in 0.1 M PBS and then fixed in ice-cold 4% paraformaldehyde for 15 min. Sections were then washed in 0.1 M PBS, followed by blocking and permeabilization for 1 h in 0.1 M PBS containing 10% goat serum, 0.03% Triton-X, and 0.1% phosphatase inhibitor (Thermo Fisher Scientific). Incubation with the primary antibodies, 4 G8 (for Aβ from Covance, Princeton Township, NJ, USA), phosphorylated tau (Ser 202, Pierce Biotechnology, Rockford, IL, USA) CREB, phosphorylated CREB (Ser 133) and NeuN (Millipore, Billerica, MA, USA), in 0.1 M PBS containing 10% serum and 0.1% phosphatase inhibitor was carried out overnight at room temperature. Following washing, slides were incubated for 1 h with Alexa Fluor secondary antibodies (Invitrogen, Carlsbad, CA, USA) in 0.1 M PBS containing 10% serum and 0.1% phosphatase inhibitor. Slides were rinsed twice in PBS and once in distilled water before a 10 min incubation with 0.3% Sudan Black B (EMD Chemicals, Gibbstown, NJ, USA) in 70% ethanol to block lipofuscin autofluorescence [62]. After rinsing in distilled deionized water, Vectashield containing 4′,6-diamidino-2-phenylindole (DAPI) was applied (Vector Laboratories, Burlingame, CA, USA), and coverslips were mounted and nail polish was used to seal the edges.

### Microscopy

High-resolution images (1024x1024 TIFF) were acquired using a confocal laser-scanning module (Bio-Rad Radiance 2000 with LaserSharp software, Hercules, CA, USA) mounted on a Nikon E800 upright microscope. For these analyses the 20x//0.75NA, 40x/0.95NA and 60x-oil/1.4NA objectives were used (Nikon, Melville, NY, USA) Images were acquired with a blue diode and krypton lasers of 488 nm and 568 nm excitation. Images for comparisons were acquired with constant settings for laser power, detector gain, amplification gain, and offset.

### Bielschowsky stain

The modified Bielschowsky stain was performed as described previously as a component of the neuropathological assessment [60].

### Aβ 1–42 ELISA assay

The soluble fraction used for this assay was generated using 200–300 mg of mid-hippocampus. The tissue was homogenized using a 1 ml syringe with a 20 gauge needle in 50 mM Tris–HCl buffer (pH = 7.6) with 0.01% NP-40, 150 mM NaCl, 2 mM EDTA, 0.1% SDS, 1 mM phenylmethylsulfonyl fluoride and protease inhibitor cocktail (Sigma-Aldrich, St. Louis, MO, USA). The homogenate was centrifuged for 5 min at 3000 rpm at 4°C and the supernatant collected for analysis [10]. Samples were normalized for protein content and diluted 1:100 in Standard Diluent Buffer provided in the ELISA kit (Invitrogen). The ELISA was performed according to the manufacturer’s directions. All samples were measured in duplicate at 450 nm.

### Generation of Aβ oligomers

To prepare oligomeric Aβ, lyophilized Aβ aliquots (0.3 mg) were dissolved in 0.2 ml 1,1,1,3,3,3-hexafluoro-2-propanol (HFP) and then added to 0.7 ml H_2_O in Eppendorf tubes. Tubes were loosely capped and samples stirred on a magnetic stirrer under a fume hood for 48 h and then used within 36 h. Quality of Aβ preparations was routinely checked by Western blot employing both NU4 and 6E10 antibodies.

#### Synaptic Fractionation

Synaptic fractionation was performed as described previously [50,63]. Briefly, hippocampal tissue was homogenized using a ground glass homogenizer and synaptosomes were isolated using a sucrose gradient and ultracentrifugation (100,000 x g for 3 h at 4°C). Synaptic junctions were obtained by incubating the synaptosomes in pH = 6 buffer (1 M Tris in 0.1 mM CaCl_2_) and then centrifuging at 40, 000 x g for 30 min at 4°C. The supernatant (containing synaptic vesicles) and the pellet were collected separately. The pellet was solublized and incubated in pH = 8 buffer (20 mM Tris, 1% Triton X-100 in 0.1 mM CaCl_2_) and then centrifuged at 40,000 x g for 30 min at 4°C to generate the PSD pellet. This pellet was solublized in 1% SDS. The supernatant containing the synaptic vesicles was concentrated using the 50 k cut-off Ampicon centrifugation tubes from Millipore followed by precipitation using acetone and then solublized in 1%SDS.

### Immunoblotting

Primary antibodies used were synaptophysin (Millipore), PSD95 (Cell Signaling, Danvers, MA, USA), and ZnT3 (LifeSpan Biosciences, Seattle, WA, USA) The monoclonal antibody 6e10 from Covance was used to detect Aβ. The Aβ oligomer-specific antibody, NU4 (a gift from the laboratory of William L. Klein), was used to detect LMW oligomers. Ponceau S staining of the membranes was performed to ensure equal protein loading before incubation with antibodies. In addition, tubulin or GAPDH (Cell Signaling) was used as a loading control. After incubation with appropriate HRP-conjugated (Sigma-Aldrich) or fluorescent secondary antibody (1:10,000) (LI-COR Biosciences, Lincoln, NE, USA), the membrane was either imaged after ECL incubation or scanned directly by an Odyssey infrared fluorescent imaging system (LI-COR Biosciences). Band densities were analyzed using Quantity One (Bio-Rad) or Odyssey software and normalized using the measured loading control densities. Each group was represented on each blot. Differences between groups were determined using a one-way ANOVA followed by Tukey post-hoc analysis.

Different loading controls were utilized depending on the sample fraction. This approach was necessary because many housekeeping proteins such as tubulin and GAPDH have altered expression and localization in aging and AD [64,65]. A variety of housekeeping proteins and total protein stains [66] were tested in preliminary experiments to determine the most appropriate loading control for each fraction. The loading control whose levels did not change depending on disease state was used for that particular protein fraction.

### Methods for the analysis of Zn^2+^

All laboratory processing was performed under clean contaminant free conditions, to minimize external metal contamination. Each sample was evaporated to dryness in a drying oven at 80 ^0^ C. Digestion of the residue was carried out with a well established procedure [67] using 0.5 mL of 30% hydrogen peroxide (GFS Chemicals, Powell, OH, USA) at 70 ^0^ C for 18–24 h followed by 0.10 mL of Ultra-pure nitric acid (GFS Chemicals) until completely ashed. The digested white ash was dissolved in 4.0 mL of Milli-Q deionized distilled water. Each digested sample was further suitably diluted 1:20, 1:40, 1:50, 1:80, 1:100 or 1:200 (v/v) using Milli-Q deionized distilled water prior to analysis.

Concentrations of Zn^2+^ in the diluted digested samples were determined by GF-AAS as described previously [63]. A Varian Instruments Model-240Z Zeeman atomic absorption spectrophotometer (Varian, Inc., Walnut Creek, CA, USA) equipped with a Varian GTA-120 graphite tube analyzer, a PSD-120 programmable sample dispenser, a Varian UltrAA high intensity boosted hollow cathode lamp were routinely used to measure Zn^2+^ at low parts per billion (ppb) levels in solution. Pyrolytically coated Varian graphite partition tubes were used for all GF-AAS analyses. Argon gas (0.3 L/min flow) was used to protect and purge the graphite tubes during the furnace program steps, and the data acquisitions were carried out using Varian SpectrAA software.

The auto-sampler cups were washed initially with 0.5 N Ultra-pure nitric acid followed by Milli-Q deionized distilled water prior to use. Calibration standards (0.0, 0.5, 1.0, 1.5, and 2.0 micrograms per liter (ppb)) of Zn^2+^ were prepared in Milli-Q deionized distilled water and run with each set of samples and the absorbance was measured at 213.9 nm wavelength. Precision and accuracy of the method were routinely checked by digesting known weights of SRM1577a standard liver powder (NIST, Bethesda, MD, USA) and analyzing them routinely along with these samples. These liver powders contain known certified amounts of several metals including Zn^2+^.

### Data collection and statistical analysis

For immunohistochemistry, analysis was performed with ImageJ (NIH) and a set of plug-ins developed by Wright Cell Imaging Facility (http://www.uhnres.utoronto.ca/facilities/wcif/imagej/). Two images per region (DG and CA3) were analyzed for each clinical case. A blind counter quantified the number of neurons in the field of view, and how many of these exhibited robust nuclear pCREB immunoreactivity. This value is expressed as the percentage of neurons that are pCREB positive. Microsoft Excel was used for graph production and StatPlus or Sigmaplot were used for data analysis. One-way ANOVA with a Fisher post hoc test was used to determine statistical significance. If the data failed the normality test (Shapiro-Wilk), a Bonferroni correction was made before statistical analysis. Data represents mean ± SEM.

## Abbreviations

Nuclear factor of activated T-cells: NFAT; 2-amino-3-(5-methyl-3-oxo-1,2- oxazol-4-yl)propanoic acid: AMPA; N-methyl-D-aspartate: NMDA; National Institutes of Health: NIH; Clinical Dementia Rating: CDR; CERAD: Consortium to Establish a Registry for Alzheimer’s Disease.

## Competing Interests

The authors declare no competing interests.

## Author’s contributions

NLB preformed synaptic fractionation and immunoblotting. LCR and VG preformed immunohistochemistry. V-MS preformed all metal analyses. RLW did the neuropathological analysis, provided tissue samples and case information. GT conceived of and supervised project, prepared manuscript along with NLB, LR, V-MS and RLW. All authors read and approved the final manuscript

## Supplementary Material

Additional file 1 **Phosphorylated CREB levels are not significantly altered when detected by Western blot in hippocampal total homogenates.** (A) Representative Western blot of total and phosphorylated CREB in hippocampal total homogenate fractions prepared from control, AD and NDAN cases. Densitometric analysis shown in (B) revealed only a trend of reduced pCREB levels in the AD samples, which however did not reach statistical significance (ANOVA, *p* > 0.05); n = 6 per group. Click here for file

Additional file 2 **Variability in postmortem interval does not correlate with differences in protein and Zn**^**2+**^**measurements performed.** Abbreviations are as follows; SF – soluble fraction, PSD – postsynaptic density, and SV – synaptic vesicles. A Pearson correlation test was preformed for each measurement against the postmortem interval. Each correlation coefficient (r) and p value is noted in the plot. Click here for file
