# Supplementary figures and images for: Absence of amyloid β oligomers at the postsynapse and regulated synaptic Zn2+ in cognitively intact aged individuals with Alzheimer’s disease neuropathology
Source: Mol Neurodegener. 2012 May 28;7:23. doi: 10.1186/1750-1326-7-23 (PMC3403985; doi:10.1186/1750-1326-7-23)

Additional file 1

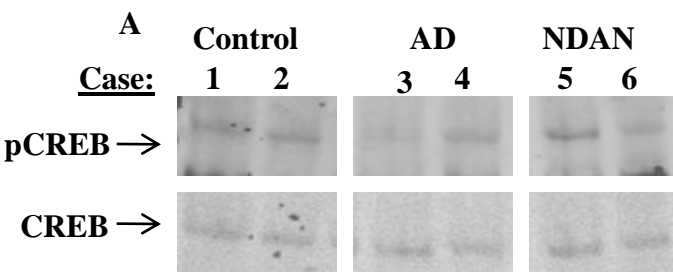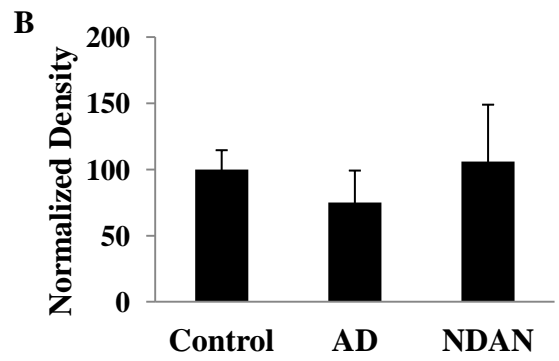

Supplement: Additional file 1 — Phosphorylated CREB levels are not significantly altered when detected by Western blot in hippocampal total homogenates. (A) Representative Western blot of total and phosphorylated CREB in hippocampal total homogenate fractions prepared from control, AD and NDAN cases. Densitometric analysis shown in (B) revealed only a trend of reduced pCREB levels in the AD samples, which however did not reach statistical significance (ANOVA, p > 0.05); n = 6 per group. [file 1750-1326-7-23-S1.pdf]

Additional file 2

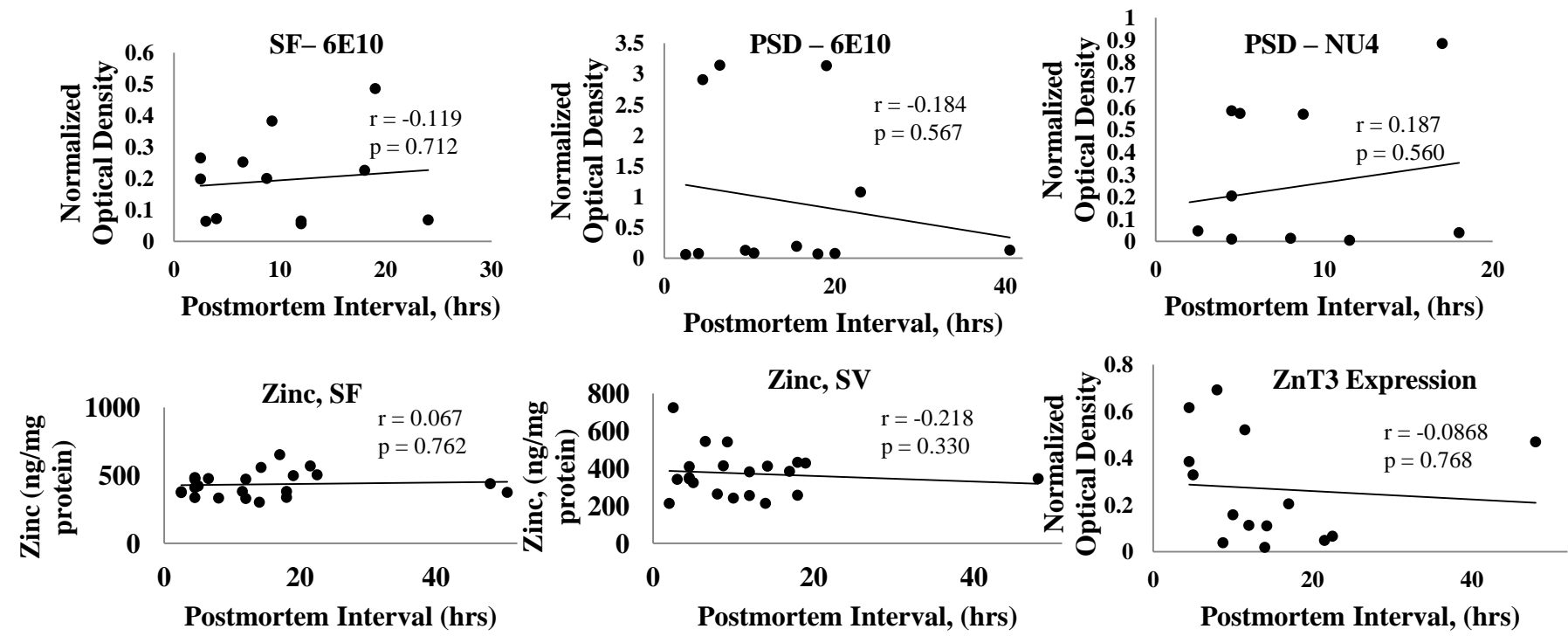

Supplement: Additional file 2 — Variability in postmortem interval does not correlate with differences in protein and Zn2+measurements performed. Abbreviations are as follows; SF – soluble fraction, PSD – postsynaptic density, and SV – synaptic vesicles. A Pearson correlation test was preformed for each measurement against the postmortem interval. Each correlation coefficient (r) and p value is noted in the plot. [file 1750-1326-7-23-S2.pdf]
